# Profiling the Nutritional, Phytochemical, and Functional Properties of Mung Bean Varieties

**DOI:** 10.3390/foods14040571

**Published:** 2025-02-08

**Authors:** Fekiya Mohammed Idris, Kelbessa Urga, Habtamu Admassu, Eskindir Getachew Fentie, Sook-Min Kwon, Jae-Ho Shin

**Affiliations:** 1 Center for Food Science and Nutrition, College of Natural Sciences, Addis Ababa University, Addis Ababa 16417, Ethiopia; mfekiya@yahoo.com; 2 Department of Chemical Engineering, Food Process Engineering Program, College of Engineering, Addis Ababa Science and Technology University, Addis Ababa 16417, Ethiopia; eskench@gmail.com; 3 Biotechnology and Bioprocess Center of Excellence, Addis Ababa Science and Technology University, Addis Ababa 16417, Ethiopia; 4 Ethiopian Public Health Institute, Ministry of Health, Addis Ababa 1242, Ethiopia; kelbessau@yahoo.com; 5 NGS Center, Kyungpook National University, Daegu 41566, Republic of Korea; 6 Department of Integrative Biotechnology, Kyungpook National University, Daegu 41566, Republic of Korea; ksv6178@gmail.com; 7 Department of Applied Biosciences, Kyungpook National University, Daegu 41566, Republic of Korea

**Keywords:** mung bean, lysine, polyphenol, rheology, antioxidant activity, cultivars

## Abstract

The Ethiopian Agricultural Research Institute (EARI) adopted four mung bean varieties for cultivation, following extensive research on their adaptability, productivity, and drought tolerance. However, the physicochemical, techno-functional, and antioxidant properties of these cultivars, which can vary significantly due to genetic and agro-ecological differences, have not been sufficiently explored in previous research. Hence, this study aimed to elucidate these properties to facilitate their seamless integration into food formulation and product development. The study results revealed that the protein content of these varieties ranged from 22.63 to 25.84 g/100 g, while carbohydrate content ranged from 54.9 to 58.82 g/100 g. Moreover, all examined varieties exhibited elevated levels of essential amino acids, particularly lysine, phenylalanine, and leucine. The foaming capacity and emulsion activity varied between 40.27–49.2% and 52.75–54.13%, respectively. The loss modulus of all varieties surpassed the storage modulus. Total polyphenol and flavonoid contents ranged from 2.36 to 3.05 mg GAE/g and 1.42 to 2.22 mg QE/g, respectively. The antioxidant activities were assessed using different assays and revealed that all samples were concentration-dependent, with all cultivars exhibiting high antioxidant activity at higher concentrations. The comparative analysis of the examined varieties revealed that none excelled in all of the tested parameters. However, these diverse qualities make Ethiopian mung bean varieties suitable for various food formulations tailored to specific desired characteristics.

## 1. Introduction

Mung beans (*Vigna radiata* L.), native to India, are a highly nutritious and economically valuable legume crop that has gained prominence across East, South, and Southeast Asia, as well as parts of Africa [1]. Renowned for their exceptional nutritional profile, mung beans are an excellent source of plant-based protein, dietary fiber, iron, folate, and essential vitamins such as B-complex vitamins. They also provide substantial amounts of macro- and micronutrients, including magnesium, potassium, zinc, and copper, alongside bioactive phytochemicals like phenolic acids, flavonoids, and saponins [2]. These bioactive compounds contribute to several health benefits, including antioxidant, anti-inflammatory, and anti-tumor activities, making mung beans a critical food resource for populations facing nutritional challenges [3,4,5].

In addition to their nutritional significance, mung beans are valued for their agronomic traits, including a short growth cycle of about 60 days, high drought tolerance, and adaptability to semi-arid and arid environments, which makes them suitable for sustainable cultivation in water-limited regions [6]. In Ethiopia, mung beans have recently emerged as a promising crop, cultivated on approximately 41,633 hectares and yielding 1.235 tons per hectare, with an annual production of 514,227 quintals. To support agricultural productivity, the Ethiopian Agricultural Research Institute (EARI) introduced four mung bean varieties: Rasa (N-26), Borda (MH-97-6), NVL-1, and Arkebe (SML-668). These varieties were developed based on adaptability, productivity, and drought resistance. Notably, while most varieties originated in India, Rasa (N-26) was sourced from Kenya [7,8].

Mung beans are not only a significant dietary component due to their protein, iron, and folate content but are also a source of essential amino acids, dietary fiber, fatty acids, and bioavailable minerals. Their phytochemical composition—including flavonoids, phenolic acids, and tannins—enhances their role in reducing oxidative stress, improving metabolic health, and supporting immune function [3,4,5]. However, mung bean’s nutritional, functional, and antioxidant activity varies among and within cultivars due to genetic, environmental, and post-harvest factors [8,9]. Despite their nutritional and agronomic potential, detailed studies on Ethiopian mung bean cultivars remain limited. Previous research has primarily focused on yield improvement, uniform maturity, and resistance to environmental stressors. To fully integrate these varieties into the food value chain and leverage their health benefits, investigations into their physicochemical, techno-functional, and health-promoting properties are essential. This study addresses these gaps by analyzing the nutritional composition, techno-functional properties, and antioxidant activity of four mung bean varieties cultivated in Ethiopia.

## 2. Materials and Methods

### 2.1. Raw Materials Collection, Preparation, and Storage

Four varieties of mung bean seeds, namely, Rasa (N-26), Baroda (MH-97-6), NVL-1, and Shoarobit (local), were obtained from Melkasa Agricultural Research Center, Ethiopia. The seeds were then cleaned, ground, and sieved through 400 μm mesh before being stored at a refrigerated condition of 4 °C for future analysis.

### 2.2. Proximate and Mineral Analysis

The proximate composition of mung bean varieties, including moisture content, crude protein, fat, ash, and fiber was determined by following the standard procedure of AOAC [10]. Furthermore, the mineral contents were analyzed by following the methods developed by Schultz et al. [11] using inductively coupled plasma atomic emission spectroscopy (Spectro CIROS ICP–AES, Spectro Analytical Instruments, Kleve, Germany).

### 2.3. Amino Acid Profiles

The samples were first hydrolyzed with 6 M HCl overnight and derivatized with 30 µL of MTBSTFA and 100 µL of acetonitrile at 100 °C for 1 h, before being injected into a GC-MS system (Thermo Fisher Scientific, Waltham, MA, USA) with a 10:1 split ratio program. The separation was achieved using a non-polar Rxi-5Sil MS capillary column (30 m length, 0.25 mm ID, 0.25 µm film thickness) (Restek, Bellefonte, PA, USA) with helium as the carrier gas at a 1 mL/min flow rate. The oven temperature was initially set at 80 °C for 1 min, ramped to 170 °C at a rate of 10 °C/min, held for 1 min, and then increased to 320 °C at a rate of 30 °C/min for 3 min. The mass spectrometer operated in electron impact (EI) mode at an ionization energy of 70 eV, scanning mass-to-charge ratios (*m*/*z*) from 30 to 700.

### 2.4. Color Analysis

A spectrophotometer (V-770 UV-Visible, Tokyo, Japan) was used to measure the color of mung bean flour, following a baseline scan from 200 to 800 nm. The specular component was included in both sample and reference measurements, and six spectra were obtained to assess measurement reproducibility by varying the measurement position. The Spectra Manager software determined the L*, a*, and b* color coordinates.

### 2.5. Phytochemical Contents

Following effective sample extraction using methanol as solvent, total polyphenol content (TPC) was determined using Folin–Ciocalteu assay as described by Phuyal et al. [12]. The total flavonoid content (TFC) was measured following the method used by Priti et al. [13], while phytic acid was determined using the colorimetric method outlined by Shi et al. [14]. Similarly, total tannin content was analyzed using the modified vanillin–HCl in methanol as described by Khandelwal et al. [15], and oxalate content was determined following the procedure described in AOAC [10].

### 2.6. Techno-Functional Properties

The water-absorption capacity (WAC), oil-absorption capacity (OAC), emulsion activity (EA), and emulsion stability (ES) of mung bean flour were determined using the method described by Argel et al. [16]. Solubility (S) and swelling power (SP) were measured following the protocol used by Yu et al. [17]. The foaming capacity (FC) and foaming stability (FS) were evaluated using the technique described in Vinayashree and Vasu [18]. Bulk density (BD) and tapped density (TD) were measured according to the methods of Ahmed et al. [19] and Andrabi et al. [6], respectively. The falling number (FN) was determined using a Perten Falling Number Instrument (version Falling Number^®^ 10001.0 E.N., Hagersten, Sweden), following ICC Standard No. 107/1 (2000). The water activity of the mung bean flour was measured using an Aqua Lab 4TE. Similarly, the thermal properties of mung bean flour were evaluated using a differential scanning calorimeter (SKZ, 1052B, Beijing, China) equipped with a nitrogen gas intercooler following the method of Aprodu et al. [20]. The pasting properties of mung bean flour were evaluated using a Rapid Visco-Analyzer (RVA-4500, Perten, Macquarie Park, NSW, Australia) according to the AACC [21] method No. 76-21. While dynamic rheological properties were determined using a Modulator Compact Rheometer (Anton-Paar, Graz, Austria) following the method of Zhong et al. [22].

### 2.7. Determination of Antioxidant Capacity

The antioxidant activity of mung bean flour was evaluated using DPPH, Ferric Reducing Antioxidant Power (FRAP), and ABTS•+ radical cation scavenging activity assays by following the methods used by Wathoni et al. [23], Xie et al. [24], and Lee et al. [25], respectively.

### 2.8. Statistical Analysis

Every data set was acquired in triplicate and presented as mean ± standard deviation. After checking the normality and homoscedasticity of the data, the statistically significant differences (*p* < 0.05) were assessed through parametric one-way analysis of variance (ANOVA). Duncan’s multiple range tests were employed as post-hoc tests to compare between sample means. All statistical data analyses were performed using R software version 4.2.3.

## 3. Results and Discussion

### 3.1. Proximate Composition

**Figure 1:**
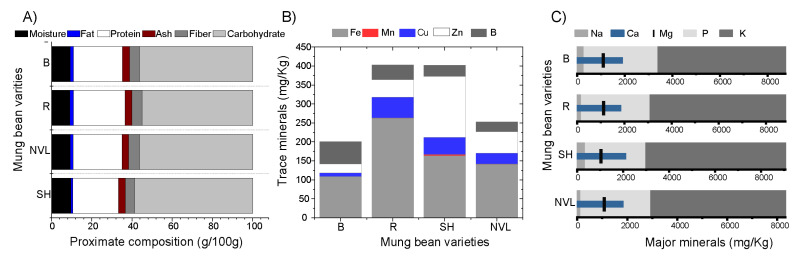
Ethiopian mung bean varieties flour (**A**) proximate composition, (**B**) trace minerals, and (**C**) major minerals composition. SH, R, B, and NVL are the mung bean variety’s name codes representing Shoarobit, Rasa, Baroda, and NVL-1, respectively.

Protein and carbohydrates are the two most dominant nutritional components of mung bean seed (Figure 1A). The crude protein content of mung bean varieties under the current investigation ranged from 22.63 to 25.84 g/100 g. This value is comparable to that of other legumes, such as soybeans (ranging from 25.5% to 36.62%), which are renowned for their high protein content [23]. Like other legumes, mung bean varieties showed a lower fat content ranging from 1.13 to 1.63 (g/100 g) (Figure 1A). Van Hung et al. [24] also reported a similar low-fat (1.6%) content for the mung bean variety. The Rasa mung bean variety exhibited significantly higher protein and fat content (*p* < 0.05) compared to the other Ethiopian mung bean varieties. This variation could be attributed to the genetic differences of this particular variety, which was adapted from Kenya, whereas the other varieties originated from India and were cultivated in Ethiopia. The carbohydrate contents of the studied mung bean varieties ranged from 54.9 to 58.82 g/100 g (Figure 1A). Notable differences were observed, except for the Baroda and NVL varieties. There was no significant difference (*p* < 0.05) in moisture content recorded for the studied mung bean varieties (9.17% to 9.5%) (Table S1). The same harvesting, storage, and preparation process for all sample varieties might contribute to the abovementioned result. Moreover, the ash content in the current study ranged from 3.33 to 3.67 (g/100 g) (Figure 1A). The Baroda variety, in particular, showed a significantly (*p* < 0.05) higher ash content than the other varieties (Table S1).

Wang et al. [9] reported a comparable ash content result for mung bean varieties collected from different regions of China. Generally, based on the proximate composition results of this study, Ethiopian mung bean varieties can be categorized as a nutritious and versatile ingredient suitable for various food products including but not limited to the development of meat analogs [23].

### 3.2. Mineral Composition

This investigation revealed that Ethiopian mung bean varieties are a significant source of major and trace minerals (Figure 1B,C). A similar investigation conducted by [26] reported that mung beans are abundant sources of major and trace minerals including Ca, Mg, Zn, Fe, P, and K. Major and trace minerals contribute significantly to overall health, from bone growth and structure to muscle contraction, nerve impulse transmission, energy metabolism, immune system function, and antioxidant defenses [27].

The mineral content of the mung bean varieties cultivated in Ethiopia showed significant differences (*p* < 0.05) among each other (Figure 1B,C). The Shoarobit variety for instance demonstrated a significantly higher (*p* < 0.05) calcium content (2095.03 mg/kg), while the Baroda variety showed a significantly higher (*p* < 0.05) phosphorus content (3347.30 mg/kg) when compared to the other varieties (Figure 1C; Table S2). A mung bean variety with high phosphorus content is anticipated to possess genetic traits that enhance its ability to absorb and utilize phosphorus from the soil efficiently [28]. In the case of trace minerals, a significantly higher (*p* < 0.05) iron content and a significantly lower (*p* < 0.05) zinc content were obtained for Rasa and Baroda varieties, respectively (Table S3). In general, factors such as genetic traits, soil conditions (including pH, nutrient availability, and composition), and post-harvest handling practices can influence the ability of crops to absorb minerals from their ultimate source, which is the soil they grow in. Even within the same growth location, the mineral uptake mechanism from the soil may differ among different varieties, depending on factors such as root mycorrhiza and plant architecture [29].

### 3.3. Amino Acid Profile

All mung bean varieties examined in this study were found to be excellent sources of essential amino acids, particularly lysine, threonine, phenylalanine, leucine, and histidine (Table 1). The concentrations of these essential amino acids exceed the FAO/WHO (2007) reference values [30]. The lysine content, which ranks as the second most abundant amino acid with an average ranging from 6.71 to 7.77 mg/g, sets these beans apart from other protein-rich grains. Although other grains may have higher total crude protein, they often lack essential amino acids such as lysine. Additionally, Ethiopian mung bean varieties are rich in non-essential and conditionally essential amino acids, such as glutamic acid, aspartic acid, alanine, and glycine (Table 1). In particular, glutamic acid, the most abundant amino acid, with an average value between 8.54 and 10.65 mg/g, plays a critical role in protein synthesis and facilitates communication between brain cells, contributing to memory, cognition, and mood regulation. Based on essential amino acid content, especially lysine, phenylalanine, and leucine, the NVL varieties, followed by the Rasa variety, exhibited significantly higher (*p* < 0.05) values compared to the other varieties (Table 1). This variation may be attributed to genetic differences among the varieties.

### 3.4. Color Property

The Ethiopian mung bean varieties’ color features (L*, a*, and b*) are tabulated in Table 2. The Baroda variety showed the highest a* value and L* value, indicating its lighter green color. Conversely, Shoarobit and Rasa had the lowest L* value, meaning they are the darkest mung bean flour compared to the other varieties (Table 2; Figure S1). Overall, mung bean varieties showed significant variations in color intensity and hue. This information is important for food product developers, manufacturers, and consumers since color plays a vital role in determining the quality and attractiveness of food products.

### 3.5. Phytochemicals

The TPC of the Ethiopian mung bean varieties under this investigation ranged from 2.36 to 3.05 mg GAE/g. However, Singh et al. [31] reported a higher range of TPC (14.06–31.31 mg GAE/g) for mung bean extracts. A significant difference (*p* < 0.05) was observed in the TPC values among these varieties (Table 3). Particularly Rasa has shown significantly lower (*p* < 0.05) TPC content compared to the other Ethiopian mung bean varieties. In contrast, the Baroda variety exhibited significantly higher (*p* < 0.05) TPC content compared to the other varieties. The genetic variations, and post-harvest processing methods, such as drying, storage, and cooking, might contribute to these variations [9,32]. However, it is important to understand that these factors are not mutually exclusive, and the variations in polyphenol content among mung bean varieties are likely influenced by a combination of these factors [9].

The TFC of the analyzed Ethiopian mung bean varieties ranged from 1.42 to 2.22 mg QE/g. Sutrisno [33] also reported a comparable TFC result for several varieties of mung beans, which ranged from 1.51 to 2.35 mg CE/g. Notably, the Baroda cultivar exhibited significantly higher (*p* < 0.05) TFC compared to the other varieties (Table 3). This variation may be attributed to environmental factors affecting the growth of different mung bean varieties, such as soil composition and climate. Additionally, differences in TFC values could stem from the limitations of the assay itself, as the AlCl_3_ colorimetric method does not capture all flavonoids in food components, given that some flavonoids do not react with the AlCl_3_ reagent.

The phytic acid content of mung bean varieties in this study was in the range of 3745 to 5175 (µg/gm) (Table 3). This value was in the range of data reported by Grewal and Jood [34]. Moreover, the levels of phytic acid also varied significantly (*p* < 0.05) among different mung bean varieties (Table 3). Specifically, the Rasa variety showed a significantly higher phytic acid content as compared to the other varieties. This variation may be attributed by genetic differences among these mung bean varieties, particularly variations in the synthesis and accumulation of phytic acid. At physiological pH, phytic acid (C_6_H_18_O_24_P_6_) carries a negative charge, giving it a high affinity for positively charged divalent mineral ions, thereby rendering these minerals unavailable for absorption [7]. However, it is also important to recognize that phytic acid exhibits antioxidant properties and is linked to various health benefits.

The mean tannin content of Ethiopian mung bean seeds was in the range of 597.06–767.92 (mg/100 g). These values are higher than the tannin content reported by Mubarak [35] for mung bean (330 mg/100 g). Moreover, a significant tannin content difference was also observed among varieties. More specifically, the Baroda variety exhibited a significantly higher concentration of tannins (*p* < 0.05), whereas the NVL variety exhibited a significantly lower concentration of tannins (*p* < 0.05) (Table 3). Tannin is a bitter polyphenolic compound that binds to or precipitates proteins and other organic compounds such as amino acids and alkaloids. Trypsin, chymotrypsin, lipase, and amylase may all be inhibited by these tannins, which reduces the quality of protein and impedes iron absorption [36]. Conversely, tannin content in the food matrix also has both beneficial and detrimental effects on human health by acting as an antioxidant and anti-inflammatory agent.

The oxalate content of mung bean varieties under this study was relatively lower as compared to tannin and phytic acid, which ranged from 0.35 to 0.72 mg/100 g (Table 3). The Borda variety showed a significantly higher (*p* < 0.05) and the Shoarobit variety showed a significantly lower (*p* < 0.05) oxalate content. Variations in the genetic makeup of the seeds might lead to differences in oxalate accumulation between different varieties [31]. Generally, the anti-nutritional content of the examined mung bean varieties is within acceptable limits. A daily intake of tannins below the range of 1.5–2.5 g is considered safe for consumption and does not lead to any adverse effects; however, consumption exceeding this range is associated with a decreased absorption of dietary iron [37]. Additionally, the phytate content of the mung bean varieties currently under investigation is within a safe level. An ideal phytate content for healthy consumption maybe 25 mg or less per 100 g in the diet to minimize micronutrient loss.

### 3.6. Techno-Functional Properties of Mung Bean Flour

The techno-functional properties of food materials give vital information about how the ingredients will behave during and after processing. The WAC of mung bean flour varieties under this investigation ranged from 2.12 to 2.32 (g/g). However, no significant difference (*p* < 0.05) was observed among compared varieties (Table 4). The main contributing factor to this outcome could be the lack of variance in the mung bean cultivars’ carbohydrate content. Moreover, the OAC of mung bean varieties in the present investigation ranged from 1.87 to 2.0 (g/g). Particularly, the OAC of the Rasa and Shoarobit varieties showed significantly higher (*p* < 0.05) values compared to the other varieties (Table 4). The higher crude protein content of these mung bean varieties might have contributed to this result. Moreover, OAC values in the range of 1 to 2 g/g, which correspond to our findings, have been reported for numerous dry mung beans [38].

Solubility is the ability of the food components to dissolve in water under predefined conditions [39]. The average solubility value of the mung bean varieties in this study ranged from 18.6 to 20.83% (Table 4). These results are comparable with other mung bean varieties from another country, which have been reported to range from 15.1% to 23.2% [40]. Further, a significantly higher (*p* < 0.05) solubility result was observed for Rasa varieties (Table 4). This could be attributed to the difference in the content and composition of protein among the varieties [9].

In the current study, the average bulk density value of mung bean varieties ranged between 0.62 and 0.68 g/mL, which falls within the typical bulk density range for legumes (0.536 to 0.816 g/mL) [41]. Moreover, a significantly (*p* < 0.05) higher bulk density was observed for the NVL and Rasa varieties (Table 4). The Baroda variety recorded a significantly lower bulk density compared to the other samples, making it suited for complementary food formulation [42].

The Ethiopian mung bean varieties average swelling power ranged from 7.13 g/g to 6.42 g/g (Table 4). The swelling power obtained in this study is higher than that of other pulses, such as cowpea flour, 2.65 to 2.68 (g/g), and slightly lower than that of cereals (17 to 22.6) [43]. The swelling power of Shoarobit variety flour was significantly (*p* < 0.05) higher than the rest of the mung bean varieties under this investigation. The higher carbohydrate content of the Shoarobit variety might be the most probable reason for this result. This high swelling capacity property might give it a chance to be incorporated into food systems requiring swelling, such as extruded and puffed snacks and emulsified products.

The falling number of the mung bean samples ranged from 221.67 to 242s (Table 4), which is significantly lower than the falling number of wheat, which ranges from 418 to 1197s in most studies. Moreover, a significantly (*p* < 0.05) higher falling number was observed for the Baroda and Shoarobit varieties (Table 4). A greater falling number indicates that the grain will not start to germinate easily and has low enzymatic activity [44].

The ability of the mung bean food component to facilitate emulsion formation is indicated by its emulsion activity. The average emulsion activity of Ethiopian mung bean varieties ranged from 52.75 to 54.13% (Table 4). This result is consistent with a previous report by Locali-Pereira et al. with average values of 65% [45]. Rasa and Baroda showed significantly (*p* < 0.05) higher value of EA as compared to the other varieties (Table 4). The differences in food components, more specifically protein types and quantities, are essential for the formation and stability of emulsions [46].

In addition, retaining the formed emulsion during processing and storage is also equally important while considering the techno-functional properties of food components. The average emulsion stability value of mung bean flour varieties in this study was in the range of 50.22% to 52.8% (Table 4). This result agreed with other studies that reported an ES value of 51.8% for mung bean flour [47]. However, mung bean flours have a three to four times greater ES when compared to wheat flour [48]. Conversely, the values are lower as compared to other legumes, particularly chickpeas [16]. Generally, the quantity and quality of soluble proteins in the samples have a huge effect on the ES value.

The average FC and FS values of the Ethiopian mung bean varieties ranged from 40.27 to 49.2% and 32.82 to 39.97%, respectively. This result was comparable with previous reports for mung bean varieties from another country, which revealed typical FC values of 35–55% and FS values of 80–100% [41]. Moreover, Rasa varieties showed significantly (*p* < 0.05) higher values for both FC and FS values as compared to the other varieties in this study (Table 4). The higher protein content of the Rasa variety might make a big contribution to this result. The higher hydrophobic amino acid content in proteins has been linked to improved foaming ability [49], while a flour’s fat content may help keep foam structures stable.

The average water activity of Ethiopian mung bean varieties ranged from 0.59 to 0.63 (Table 4). This result is compatible with the water activity of other crops, which was reported in the range of 0.58 to 0.62. However, no significant difference (*p* < 0.05) was observed among the varieties under the study. A similar moisture content between the varieties might be the major cause for this result.

### 3.7. Thermal Property

**Figure 2:**
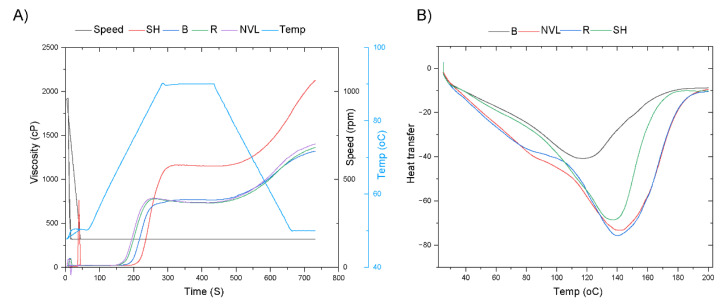
Ethiopian mung bean varieties’ (**A**) pasting properties and (**B**) differential scanning calory meter values. The abbreviations AA, SH, R, B, and NVL are used to represent ascorbic acid, and the Shoarobit, Rasa, Baroda, and NVL-1 varieties, respectively.

Thermal property analyses of food components are an important parameter to optimize cooking and other thermal processing conditions to enhance the nutrient digestibility and bioavailability of food components. The thermal properties of Ethiopian mung bean varieties were evaluated by utilizing differential scanning calorimetry (DSC) method. During the heating scan between 100 and 200 °C, the probable endothermic event in the mung seed flour might be protein denaturation (Figure 2B). The denaturation temperatures for the Baroda, Shoarobit, NVL, and Rasa cultivars were 117.2 °C, 132.8 °C, 132.7 °C, and 133.3 °C, respectively. The crude protein content, the protein profile, and molecular interactions among proteins might influence the stability of the protein structure, thereby affecting the denaturation temperature [46]. Furthermore, a Thermogravimetric analysis (TGA) was also used to evaluate the trend of mass loss as a function of temperature (Figure S3). All varieties showed significant mass loss between 200 and 600 °C.

### 3.8. Rheological Properties

#### 3.8.1. Pasting Property

The pasting property is one of the important parameters for understanding the properties of starch viscosity under a simulated cooking process. It has a significant influence on the quality and aesthetic values of the food, particularly for beaker products. The average peak viscosity (PV) of Ethiopian mung bean varieties was in the range of 771 to 11,493.33 (cP) (Figure 2A). In comparison to other cereals, particularly wheat flour (2574 cP) [44], mung bean flour has a relatively low PV. The increased protein content of legume flour in general, and mung bean flour in particular, contributed to this lower value by inhibiting starch granules not to swell [19]. Moreover, a significantly higher (*p* < 0.05) PV was observed for the Shoarobit variety (1149.33 cP) as compared to the varieties under investigation. The difference in PV values among Ethiopian mung bean cultivars could be related to the underlying structure of starch rather than total carbohydrate content.

The breakdown viscosity (BDV) of the mung bean varieties in this study was in the range of 9.67 cP to 78.67 cP (Figure 2A). This result is comparable with the BDV value (5.33 cP to 85 cP) of other legume flour in a similar study [50]. However, these values are very low as compared to the BDV of refined wheat flour [44]. The BDV is basically an indicator of starch stability as it tells the flour’s resistance to heat and shear stress during cooking. As the BDV increases, the ability of the starch to withstand heat and shear stress decreases.

The final viscosity (FV) of Ethiopian mung bean varieties was in the range of 1306 cP to 2156 cP (Figure 2A). As it compared with its peak viscosity, the FV of mung bean varieties is significantly high. This viscosity is an indication of flour’s capacity to produce a viscous paste after cooking and chilling. Additionally, it provides a measurement of the paste’s resistance to shear force when stirring [50]. A significantly higher (*p* < 0.05) FV value was recorded for the Shoarobit variety. Thus, this variety could be preferred for cooking applications, such as thickening sauces or smoothing batters, because of its increased viscosity and resistance to shear stress.

#### 3.8.2. Dynamic Rheological Properties

**Figure 3:**
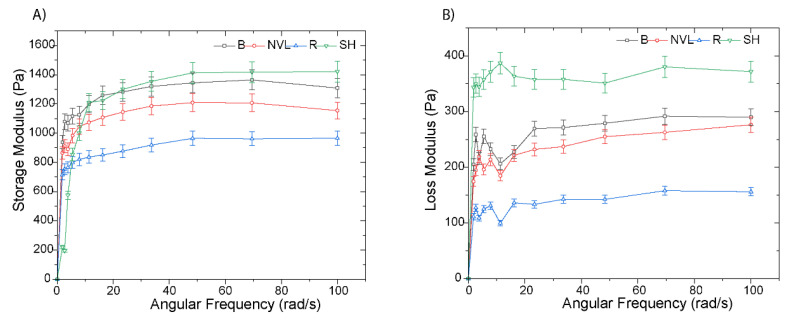
Rheological properties of mung bean varieties in Ethiopia during frequency sweep at 25 °C and 0–100 rad/sec are expressed as (**A**) storage modulus and (**B**) loss of modulus. The abbreviations SH, R, B, and NVL represent the Shoarobit, Rasa, Baroda, and NVL-1 varieties, respectively.

The dynamic rheological properties of Ethiopian mung bean varieties were evaluated by analyzing their storage (G′) and loss (G″) modulus. In all of the samples, the storage modulus was higher than the loss modulus (Figure 3). Moreover, there was no crossover between the two moduli across the complete frequency sweep range. This is an indication that the mung bean flour’s elasticity is greater than its viscosity. This combination of properties is characteristic of a weak gel, where the structure can deform under stress but retains its shape once the stress is removed [51]. Moreover, the absence of crossover between G′ and G″ further confirmed the formation of a stable gel structure in the mung bean flour paste. Their composition, primarily the starch, protein network, particle size, and particle size distribution, can be attributed to the variations in loss and storage modulus.

The loss of tangent (δ) values consistently remained below 0.25, except for the Shoarobit variety, as shown in Table 5. This indicates that the G′ value exceeded the G″ value in all of the samples, throughout the entire frequency range. The Shoarobit variety demonstrated higher storage modulus (G′) values as compared to the other varieties in this study (Figure 3). This was reaffirmed by the higher loss tangent value for the Shoarobit variety paste (0.38), suggesting a system with a greater emphasis on viscous behavior compared to the other varieties. On the other hand, the other varieties of flour pastes exhibited low tan δ values (0.15–0.21), suggesting a more organized system with a stronger solid-like behavior. The content, profile, structure, and interaction between food components, more particularly, protein and carbohydrates, contributed to the aforementioned result [52]. Generally, understanding the rheological properties of food samples will help in selecting the right mung bean variety for their specific product formulations.

### 3.9. Antioxidant Capacity

**Figure 4:**
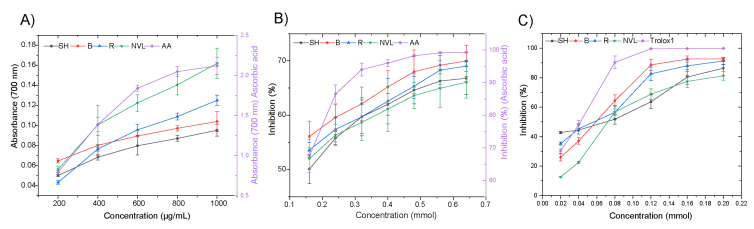
Ethiopian mung bean varieties’ antioxidant activities: (**A**) FRAP, (**B**) DPPH, and (**C**) ABTS values. The abbreviations AA, SH, R, B, and NVL represent ascorbic acid, and the Shoarobit, Rasa, Baroda, and NVL-1 varieties, respectively.

The FRAP assay evaluates the ability of a sample to reduce ferric ions (Fe^3+^) to ferrous ions (Fe^2+^). Although the reducing potential of all mung bean samples was significantly lower than that of the standard (ascorbic acid) across all concentrations, their reducing capacity increased with rising concentrations of the extracts (Figure 4A). This increase may be attributed to the presence of bioactive compounds, including polyphenols, tocopherols, carotenoids, and other metabolites. Among the varieties under this investigation, the Baroda variety exhibited a significantly higher antioxidant capacity (*p* < 0.05), specifically at a lower sample concentration. However, at higher concentrations, no significant difference in antioxidant activity was observed between the samples. Notably, at a concentration of 600 µg/mL, the NVL variety demonstrated a significantly higher antioxidant capacity compared to the other samples. The significantly higher (*p* < 0.05) TPC and TFC observed in the Baroda and NVL varieties (Table 3) may have contributed to these results.

The DPPH scavenging ability of all mung bean cultivars was concentration-dependent, with antioxidant activity increasing as the concentration increased (Figure 4B). The antioxidant activity of mung bean seeds is attributed to bioactive compounds such as proanthocyanidins, flavonoids, and lignans, which exert their effects through hydrogen bonding, hydrophobic interactions, and electrostatic interactions [5,53]. Among the varieties studied, the Baroda variety demonstrated a superior DPPH scavenging capacity, particularly at lower concentrations. However, at the final concentration (0.64 mmol), no significant differences (*p* < 0.05) were observed between the varieties. The higher levels of bioactive compounds, such as phenolic compounds and flavonoids, in the Baroda variety may explain these results. The Baroda variety’s antioxidant activity may be attributed to its unique blend of bioactive compounds, including proanthocyanidins, flavonoids, and lignans, which effectively combat free radicals and reduce oxidative stress [13,54]. The Baroda variety’s unique composition and synergistic effects may enhance its DPPH scavenging capacity and antioxidant activity, even at lower concentrations.

The ABTS radical scavenging capacity of all samples, as well as the standard Trolox (water-soluble vitamin E analog), increased with rising concentration (Figure 4C). Interestingly, at lower concentrations, the Shoarobit variety exhibited a significantly higher (*p* < 0.05) ABTS scavenging activity compared to the other samples, including the standard. This result may be attributed to the higher (next to Baroda) TPC (2.67 mg GAE/g) and TFC (1.75 mg QE/g) of the Shoarobit variety, in addition to the different mechanisms of action used in the ABTS antioxidant assay. However, at higher concentrations, the Baroda and Rasa varieties demonstrated a significantly greater (*p* < 0.05) ABTS scavenging activity when comparing between the varieties. In summary, the Baroda mung bean variety exhibited the strongest overall potential to neutralize reactive species and protect against oxidative stress across all antioxidant assays.

The results provide a substantial understanding of the antioxidant capacities of various mung bean varieties, particularly regarding concentration levels. The study indicates that the Shoarobit variety has superior antioxidant properties, possibly due to its higher total phenolic and total flavonoid content, which neutralize free radicals. The observation that the Shoarobit variety outperformed the others at lower concentrations suggests that it uses its antioxidant compounds more efficiently, making it a valuable candidate for applications where lower dosages are advantageous. This efficiency may also imply a distinctive mechanism of action that enhances its scavenging capability in the ABTS assay. In contrast, the increased ABTS scavenging activity observed in the Baroda and Rasa varieties at higher concentrations suggests that these varieties may possess a unique profile of antioxidant compounds that are more effective at elevated levels. This result indicates a threshold effect, where the antioxidant capacity of these varieties is optimized at greater concentrations, potentially due to synergistic interactions among the various phytochemicals present

## 4. Conclusions

To maximize the potential of Ethiopian mung bean varieties in diverse food applications, this study evaluated their nutritional composition, techno-functional characteristics, and antioxidant properties. As a result, notable variations were observed both among Ethiopian mung bean varieties and in comparison to mung bean cultivars from other countries. Notably, the Rasa variety exhibited a higher crude protein content, while the NVL-1 variety showed elevated levels of essential amino acids, particularly lysine, phenylalanine, and leucine. Additionally, the Baroda variety excelled in major mineral composition, while the Shoarobit variety stood out for its higher levels of trace minerals, particularly zinc—a vital micronutrient for human health. The Shoarobit variety also exhibited superior rheological properties and high thermal resistance before denaturation, making it well suited for high-temperature cooking. Furthermore, although the antioxidant activity of all varieties was concentration-dependent, the Baroda variety stood out for its higher phytochemical concentration and greater antioxidant activity. In a nutshell, these diverse attributes highlight the suitability of Ethiopian mung bean varieties for various food product developments. However, further molecular-level carbohydrate profiling and in vivo antioxidant activity studies are still required to fully explore the potential of these mung bean varieties.

## Figures and Tables

**Table 1:** Amino acid contents of mung bean seed (mg/g) varieties.

Amino Acids (mg/g)	Variety			
	SH	R	B	NVL
Histidine	2.69 ± 0.01 ^d^	3.15 ± 0.01 ^c^	3.33 ± 0.01 ^a^	3.22 ± 0.01 ^b^
Isoleucine	1.66 ± 0.01 ^d^	1.82 ± 0.01 ^a^	1.76 ± 0.01 ^c^	1.79 ± 0.01 ^b^
Leucine	2.92 ± 0.01 ^d^	3.17 ± 0.01 ^a^	2.98 ± 0.01 ^c^	3.09 ± 0.01 ^b^
Lysine	6.71 ± 0.01 ^d^	7.25 ± 0.01 ^c^	7.5 ± 0.01 ^b^	7.77 ± 0.01 ^a^
Methionine	0.68 ± 0.01 ^d^	0.71 ± 0.01 ^c^	0.82 ± 0.01 ^a^	0.79 ± 0.01 ^b^
Phenylalanine	2.93 ± 0.01 ^c^	3.2 ± 0.01 ^b^	3.34 ± 0.01 ^a^	3.35 ± 0.01 ^a^
Threonine	1.81 ± 0.01 ^b^	1.78 ± 0.01 ^c^	1.87 ± 0.01 ^a^	1.56 ± 0.01 ^d^
Valine	2.19 ± 0.01 ^d^	2.5 ± 0.01 ^a^	2.22 ± 0.01 ^c^	2.28 ± 0.01 ^b^
Alanine	2.9 ± 0.01 ^d^	3.17 ± 0.01 ^a^	2.95 ± 0.01 ^c^	2.99 ± 0.01 ^b^
Aspartic acid	3.22 ± 0.01 ^d^	3.47 ± 0.01 ^c^	3.63 ± 0.01 ^b^	3.74 ± 0.01 ^a^
Cysteine	0.29 ± 0.01 ^c^	0.31 ± 0.01 ^b^	0.52 ± 0.01 ^a^	0.31 ± 0.01 ^b^
Glutamic acid	8.54 ± 0.01 ^d^	9.74 ± 0.01 ^c^	10.65 ± 0.01 ^a^	10.34 ± 0.01 ^b^
Glycine	2.19 ± 0.01 ^b^	2.5 ± 0.01 ^a^	2.33 ± 0.01 ^b^	2.34 ± 0.01 ^b^
Proline	2.12 ± 0.01 ^d^	2.31 ± 0.01 ^a^	2.24 ± 0.01 ^c^	2.28 ± 0.01 ^b^
Serine	2.67 ± 0.01 ^c^	2.8 ± 0.01 ^b^	2.82 ± 0.01 ^a^	2.79 ± 0.01 ^b^
Tyrosine	1.55 ± 0.01 ^c^	1.67 ± 0.01 ^b^	1.75 ± 0.01 ^a^	1.68 ± 0.01 ^b^
Cystine	0.48 ± 0.01 ^b^	0.54 ± 0.01 ^a^	0.53 ± 0.01 ^a^	0.52 ± 0.01 ^a^

Different superscript letters indicate significant differences (*p* < 0.05) between the means in each column. SH, R, B, and NVL are codes representing the mung bean varieties Shoarobit, Rasa, Baroda, and NVL-1, respectively.

**Table 2:** Color measurement values of Ethiopian mung bean varieties.

Color	Variety			
	SH	R	B	NVL
L*	58.61 ± 0.04 ^c^	58.61 ± 0.03 ^c^	66.57 ± 0.04 ^a^	62.16 ± 0.04 ^b^
a*	−2.785 ± 0.01 ^a^	−2.76 ± 0.04 ^a^	−3.16 ± 0.04 ^c^	−3.07 ± 0.04 ^b^
b*	10.12 ± 0.02 ^c^	10.12 ± 0.01 ^c^	11.58 ± 0.05 ^a^	11.22 ± 0.05 ^b^

Different superscript letters indicate significant differences (*p* < 0.05) between the means in each column. SH, R, B, and NVL are codes representing the mung bean varieties Shoarobit, Rasa, Baroda, and NVL-1, respectively.

**Table 3:** Phytochemicals in mung bean flour.

Phytochemicals	Varieties			
	SH	R	B	NVL
Phytic acid (µg/gm)	3745.18 ± 5.76 ^d^	5175.19 ± 6.29 ^a^	4521.85 ± 1.05 ^b^	4264.81 ± 4.19 ^c^
Tannin (mg/100 g)	669.38 ± 2.06 ^c^	718.24 ± 3.74 ^b^	767.92 ± 0.79 ^a^	597.06 ± 2.14 ^d^
Oxalate (mg/100 g)	0.41 ± 0.04 ^c^	0.35 ± 0.04 ^c^	0.72 ± 0.05 ^a^	0.6 ± 0.04 ^b^
Total polyphenol (GAE mg/g)	2.67 ± 0.01 ^b^	2.36 ± 0.01 ^c^	3.05 ± 0.02 ^a^	2.65 ± 0.01 ^b^
Total flavonoid (QEmg/g)	1.78 ± 0.02 ^b^	1.60 ± 0.02 ^c^	2.22 ± 0.01 ^a^	1.42 ± 0.01 ^d^

Different superscript letters indicate significant differences (*p* < 0.05) between the means in each column. SH, R, B, and NVL are codes representing the mung bean varieties Shoarobit, Rasa, Baroda, and NVL-1, respectively.

**Table 4:** Techno-functional properties of mung bean flour.

Techno-Functional Properties	Varieties			
	SH	R	B	NVL
WAC (g/g)	2.24 ± 0.08 ^ab^	2.12 ± 0.08 ^b^	2.32 ± 0.05 ^a^	2.20 ± 0.07 ^ab^
OAC (g/g)	2.00 ± 0.00 ^a^	1.93 ± 0.06 ^ab^	1.89 ± 0.06 ^b^	1.87 ± 0.06 ^b^
S (%)	18.6 ± 0.42 ^b^	20.28 ± 0.35 ^a^	18.64 ± 0.31 ^b^	19.22 ± 0.39 ^b^
SP (g/g)	7.13 ± 0.32 ^a^	6.57 ± 0.08 ^b^	6.42 ± 0.38 ^b^	6.57 ± 0.14 ^b^
BD(g/mL)	0.66 ± 0.01 ^b^	0.67 ± 0.01 ^a^	0.62 ± 0.01 ^c^	0.68 ± 0.01 ^a^
TD(g/mL)	0.86 ± 0.01 ^b^	0.88 ± 0.01 ^a^	0.85 ± 0.01 ^b^	0.90 ± 0.01 ^a^
EA (%)	52.75 ± 0.16 ^c^	53.92 ± 0.08 ^a^	54.13 ± 0.28 ^a^	53.14 ± 0.20 ^b^
ES (%)	51.84 ± 0.34 ^c^	50.22 ± 0.22 ^d^	52.8 ± 0.25 ^a^	52.29 ± 0.02 ^b^
FC (%)	40.27 ± 0.46 ^c^	49.2 ± 0.35 ^a^	43.67 ± 0.58 ^b^	44.33 ± 0.58 ^b^
FS (%)	33.12 ± 1.66 ^b^	39.97 ± 1.06 ^a^	32.82 ± 1.16 ^b^	35.35 ± 1.74 ^b^
FN (Sec.)	242 ± 0.00 ^a^	236.33 ± 0.58 ^b^	242 ± 0.00 ^a^	221.67 ± 0.58 ^c^
WA	0.59 ± 0.00 ^b^	0.59 ± 0.00 ^b^	0.59 ± 0.01 ^b^	0.63 ± 0.01 ^a^

WAC, OAC, S, SP, BD, TD, EA, ES, FC, FS, FN, and WA codes represent techno-functional properties representing water-absorbing capacity, oil-absorbing capacity, solubility, swelling power, bulk density, tapped density, emulsion activity, emulsion stability, foaming capacity, foaming stability, falling number, and water activity, respectively. Different superscript letters indicate significant differences (*p* < 0.05) between the means in each column. SH, R, B, and NVL are codes representing the mung bean varieties Shoarobit, Rasa, Baroda, and NVL-1, respectively.

**Table 5:** Mean values of the storage modulus (G′), loss modulus (G″), and loss tangent (tan δ) values at 0.1–100. Rad s-1 of the flour pastes.

		Variety		
Viscoelastic Properties	SH	R	B	NVL
G′ (Pa)	1010.91 ± 2.60 ^c^	843.90 ± 6.26 ^d^	1205.89 ± 7.98 ^a^	1065.35 ± 5.95 ^b^
G″ (Pa)	385.41 ± 2.38 ^a^	132.06 ± 2.60 ^d^	255.18 ± 2.03 ^b^	222.58 ± 2.23 ^c^
tan δ (G″/G′)	0.38 ± 0.00 ^a^	0.16 ± 0.00 ^c^	0.21 ± 0.00 ^b^	0.21 ± 0.00 ^b^

Different superscript letters indicate significant differences (*p* < 0.05) between the means in each column. SH, R, B, and NVL are codes representing the mung bean varieties Shoarobit, Rasa, Baroda, and NVL-1, respectively.

## Data Availability

The original contributions presented in this study are included in the article/Supplementary Materials. Further inquiries can be directed to the corresponding authors.

## Supplementary Materials

The following supporting information can be downloaded at: https://www.mdpi.com/article/10.3390/foods14040571/s1, Figure S1: Ethiopian mung varieties (A) Baroda (MH-97-6), (B) Rasa (N-26), (C) NVL-1, (D) Shoarobit (land raised); Figure S2: Fourier transform infrared of Rasa, Shoarobit, NVL-1, and Baroda variety mung bean; Figure S3: The TGA results of Ethiopian mung bean varieties SH, R, B, and NVL are name codes that represent Shoarobit, Rasa, Baroda, and NVL-1, respectively; Table S1: Proximate composition of Ethiopian mung bean varieties; Table S2: Major mineral composition of Ethiopian mung bean varieties; Table S3: Trace mineral composition of Ethiopian mung bean varieties; Table S4: Pasting property.
