# Supplementary material for: Profiling the Nutritional, Phytochemical, and Functional Properties of Mung Bean Varieties
Source: Foods. 2025 Feb 8;14(4):571. doi: 10.3390/foods14040571 (PMC11854456; doi:10.3390/foods14040571)
Supplement: Supplementary file 1 [file foods-14-00571-s001.zip › foods-3440692-supplementary.pdf]

Supplementary Material

Table S1: Proximate composition of Ethiopian mung bean varieties

**Varieties**	**Proximate composition (g/100g)**					
	Moisture content	Fat	Protein	Ash	Fiber	Carbohydrate
SH	9.5 ± 0.05[a]	1.13 ± 0.08[c]	22.63 ± 0.03[c]	3.42 ± 0.04[ab]	4.5 ± 0.05[d]	58.82 ±0.06[a]
NVL	9.33 ± 0.09[a]	1.35 ± 0.03[bc]	24.33 ± 0.06[b]	3.33 ± 0.04[b]	5.36 ± 0.02[a]	56.3 ± 0.05[b]
R	9.17 ± 0.09[a]	1.63 ± 0.08[a]	25.84 ± 0.01[a]	3.42 ± 0.04[ab]	5.15 ± 0.09[b]	54.9 ± 0.05[c]
B	9.33 ± 0.08[a]	1.51 ± 0.06[ab]	24.62 ± 0.03[b]	3.67 ± 0.04[a]	4.88 ± 0.04[c]	56.3 ± 0.03[b]

Table S2: Major mineral composition of Ethiopian mung bean varieties

Varieties	Ca	Mg	K	Na	P
B	1888.79 ± 1.43[b]	1089.22 ± 1.40[ab]	8753.86 ± 1.54[b]	284.35 ± 1.22[b]	3347.30 ± 1.51[a]
R	1842.02 ± 1.84[c]	1121.16 ± 1.56[a]	8824.63 ± 1.83[ab]	182.99 ± 1.50[c]	3030.32 ± 2.07[b]
SH	2095.03 ± 1.81[a]	1033.51 ± 1.11[b]	9055.89 ± 1.78[a]	358.66 ± 0.95[a]	2930.19 ± 1.84[b]
NVL	1825.95 ± 1.68[c]	1082.72 ± 1.58[ab]	8332.80 ± 1.40[c]	152.29 ± 1.98[d]	2898.24 ± 1.74[b]

Table S3: Trace mineral composition of Ethiopian mung bean varieties

Varieties	Fe	Mn	Cu	Zn	B
B	108.49 ± 1.95[d]	0.52 ± 0.02[c]	9.16 ± 0.44[d]	23.30 ± 1.09[d]	58.98 ± 1.52[a]
R	262.41±1.56[a]	1.30 ± 0.20[b]	54.55 ± 1.1[a]	45.66 ± 0.40[c]	39.28 ± 0.44[b]
SH	163.60 ± 2.04[b]	3.81 ± 0.23[a]	44.39 ± 1.13[b]	160.39 ± 1.83[a]	29.83 ± 0.31[c]
NVL	141.39 ± 1.25[c]	0.47 ± 0.02[c]	28.20 ± 0.46[c]	56.47 ± 0.75[b]	26.30 ± 0.30[d]

Table S4. Pasting property

	**Varieties**			
**Parameters**	SH	B	NVL	R
**Peak 1**	1149.33 ± 28.04[a]	771.67±16.04[c]	843 ± 7.55[b]	779±7.55[c]
**Trough 1**	1135.33 ± 28.01[a]	762.00 ± 14.53[b]	764.33 ± 4.51[b]	730 ± 6.00[c]
**Breakdown**	14 ± 1.00[c]	9.67 ± 1.53[c]	78.67 ± 6.35[a]	49 ± 1.73[b]
**Final peak**	2156 ± 94.32[a]	1306.33 ± 28.04[c]	1449.67 ± 16.17[b]	1390.67 ± 23.18[bc]
**Setback**	1020.67 ± 68.3[a]	544.33 ± 41.24[c]	685.33 ± 11.68[b]	660.67 ± 24.44[b]
**Peak time**	5.67 ± 0.12[b]	6.58 ± 0.24[a]	4.78 ± 0.04[c]	4.98 ± 0.10[c]
**Pasting Temp**	78.98 ± 0.41[b]	81.37 ± 0.85[a]	78.95 ± 0.43[b]	79.15 ± 0.43[b]

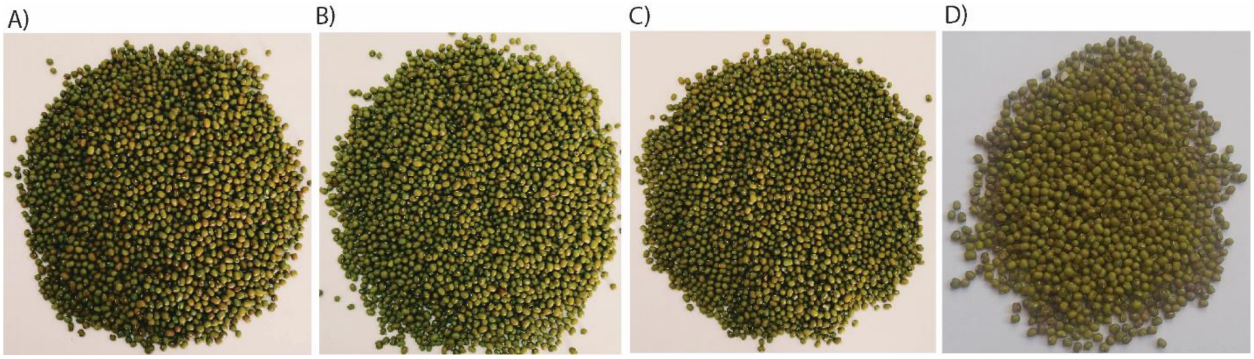

Figure S1 Ethiopian mung bean varieties A) Baroda (MH-97-6), B) Rasa (N-26), C) NVL-1 D) Shoarobit (land raised)

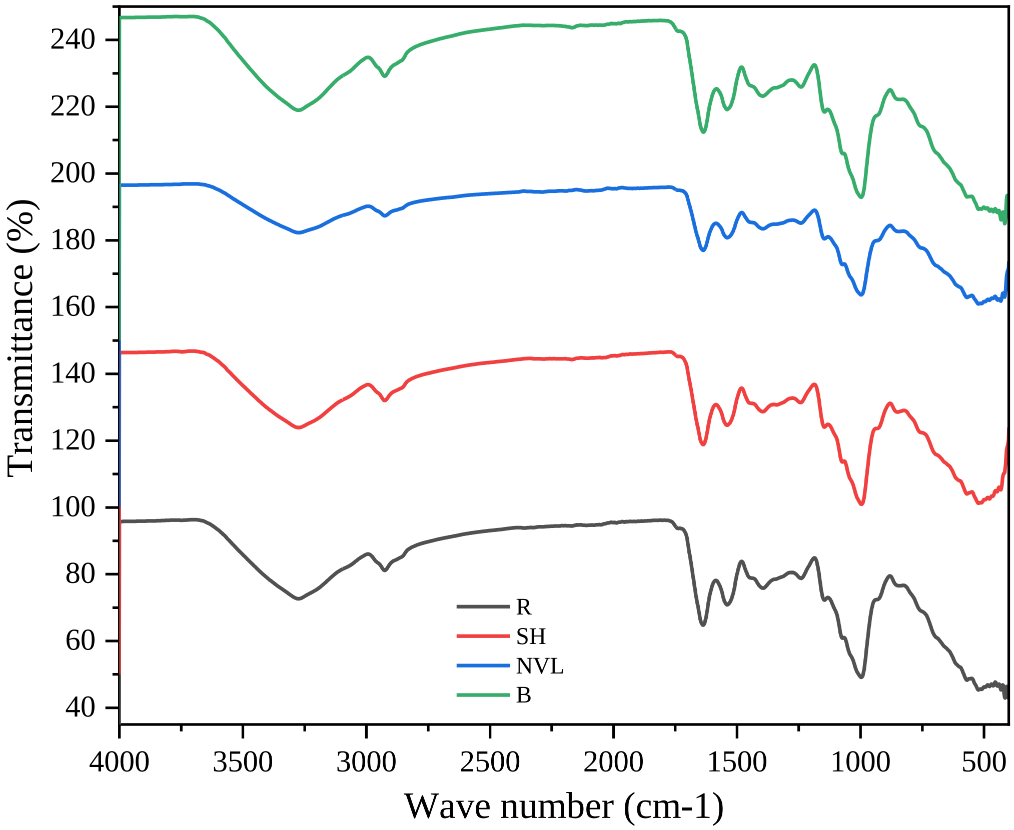

Figure S2. Fourier transform infrared of Rasa, Shoarobit, NVL-1, and Baroda variety mung bean.

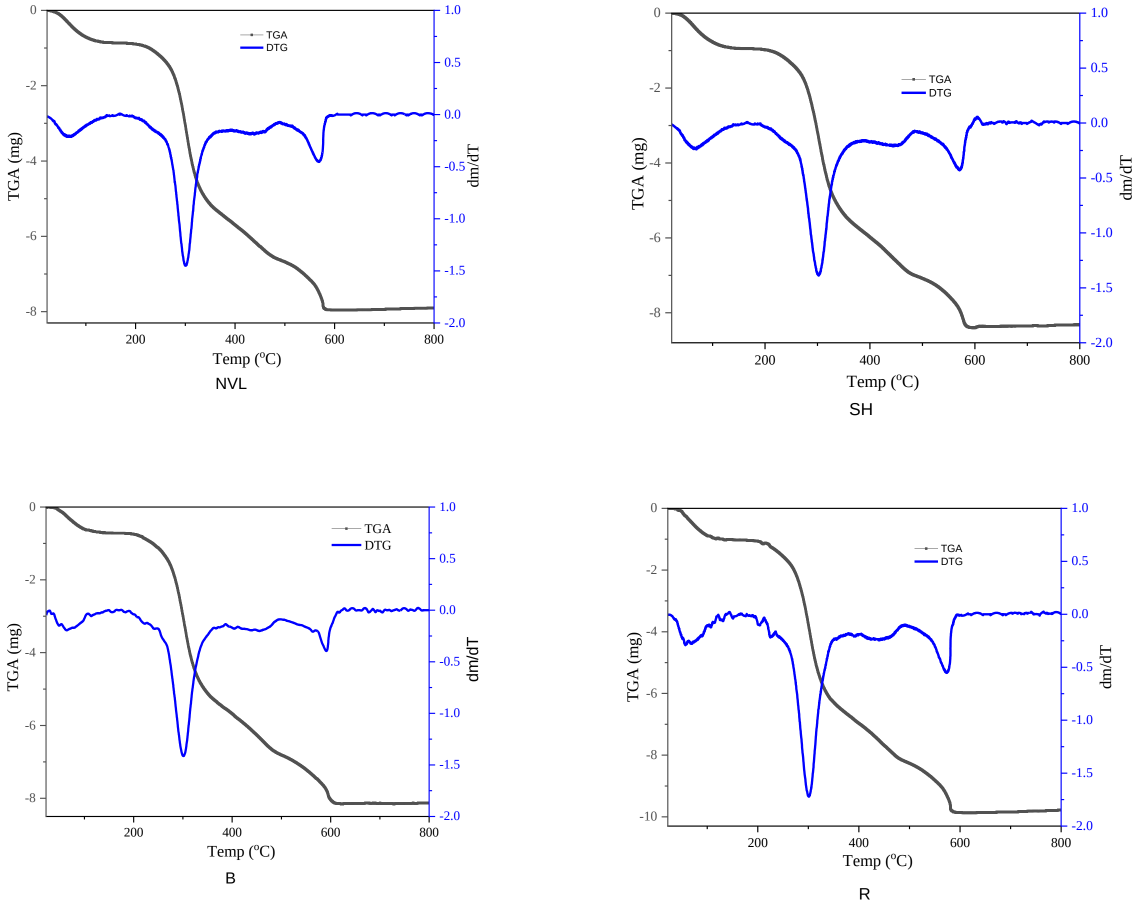

Figure S3. The TGA results of Ethiopian mung bean varieties SH, R, B, and NVL are name codes that represent Shoarobit, Rasa, Baroda, and NVL-1, respectively.
